# Bad situation, treat yourself: a qualitative exploration of the factors influencing healthy eating habits during the COVID-19 pandemic

**DOI:** 10.1080/21642850.2023.2182307

**Published:** 2023-03-02

**Authors:** Christine A. Pellegrini, Katherine DeVivo, Andrea T. Kozak, Jessica L. Unick

**Affiliations:** a Department of Exercise Science, Arnold School of Public Health, University of South Carolina, Columbia, SC, USA; b Department of Psychology, Oakland University, Rochester, MI, USA; c The Miriam Hospital’s Weight Control and Diabetes Research Center, Warren Alpert Medical School at Brown University, Providence, USA

**Keywords:** Diet, obesity, behavioral weight management, COVID-19

## Abstract

**Purpose:**

To explore barriers and facilitators to healthy eating during the COVID-19 pandemic among adults enrolled in an internet-based weight loss program.

**Methods:**

Adults in an internet-delivered weight loss program were recruited to participate. Participants completed online study surveys and a semi-structured interview via telephone between June 1, 2020 and June 22, 2020. The interview included questions to explore how the COVID-19 pandemic has influenced dietary behaviors. Constant comparative analysis was used to identify key themes.

**Results:**

Participants (*n* = 30) were primarily female (83%) and white (87%), 54.6 ± 10.0 years old, and had a mean body mass index of 31.1 ± 4.5 kg/m^2^. Barriers included snacking/ease of access to food, eating as a coping mechanism, and lack of routine/planning. Facilitators included calorie control, regular routine/scheduling, and self-monitoring. General themes with eating were a change in eating out frequency or modality, cooking more, and changes in alcohol consumption.

**Conclusion:**

Eating habits among adults enrolled in a weight loss program changed during the COVID-19 pandemic. Future weight loss programs and public health recommendations should consider modifying recommendations to place increased emphasis on strategies to overcome barriers to healthy eating and promote facilitators that may help with healthy eating, particularly during unexpected circumstances or events.

## Introduction

The World Health Organization declared Coronavirus Disease 2019 (COVID-19) a pandemic (WHO Director-General’s opening remarks at the media briefing on COVID-19 - 11 March, 2020) and following the declaration of a National Emergency on March 11, 2020, actions were taken to slow the spread of the disease in the United States. The social distancing and safety precautions recommended led to closures, stay-at-home orders, and a sudden shift to e-learning and working from home. As a result, the population’s day-to-day activities and normal routines were disrupted.

With the abrupt changes in daily life, many studies have suggested that dietary quality was lower in the peak lockdown phase of the pandemic (Bennett et al., 2021; Mitchell et al., 2021). Further, some studies have suggested that alcohol consumption changed during this time, either increasing (Pollard et al., 2020; Tran et al., 2020) or decreasing, (Chodkiewicz et al., 2020) potentially depending on initial consumption (Rossow et al., 2021). During this time, many adults also experienced weight gain (Bhutani et al., 2021; Seal et al., 2022). Given that over 70% of Americans meet criteria for overweight/obesity, changes in diet, alcohol consumption, and weight are concerning and may put these individuals at elevated risk for early mortality and the development of chronic conditions.

While many studies have reported lower dietary quality during the pandemic, (Bennett et al., 2021; Mitchell et al., 2021) few studies have examined the factors that may have influenced eating behaviors, (Silva et al., 2021) particularly among those actively trying to lose weight. Therefore, this qualitative study explored eating behaviors during the COVID-19 pandemic among participants enrolled in an internet-delivered weight loss program. Specifically, this study explored factors that may have been helping or hindering healthy eating behaviors in June 2020 when initial stay-at-home orders were being lifted, social gatherings were limited to 15 people, outdoor dining was allowed, and offices could have one-third of employees return to work in-person. Gaining a better understanding of the factors that influenced eating behaviors would allow for the tailoring of weight loss programs to promote identified facilitators and problem solve against recognized barriers that may occur during unforeseen circumstances, stressful life situations, or periods of major life change.

## Materials and methods

### Participants

Individuals who were actively participating in a larger, randomized trial through a hospital-based research center which included an internet-delivered behavioral weight loss program, were recruited for the current qualitative study via an email invitation. Participants were recruited from the first 3 cohorts and had been enrolled for 4, 7, or 11 months. The purpose of the larger randomized clinical trial was to examine whether providing brief or extended phone coaching to those with sub-optimal early weight loss during the internet-delivered program improved weight loss outcomes. All participants received a 12-month internet-delivered program and were given a 10% weight loss goal. To facilitate this weight loss, participants were given a calorie intake goal between 1200 and 1800 kcal/day, asked to self-monitor daily caloric intake and encouraged to engage in 150 min/week of moderate-intensity physical activity. Computer-generated feedback based on self-monitoring data was provided and video lessons were available on the website which covered topics such healthy eating, physical activity, and behavioral strategies. The study eligibility criteria for the weight loss program has been published previously, (Unick et al., 2020) but in short, participants were 18–70 years old with a baseline body mass index between 25 and 45 kg/m^2^ and resided in Rhode Island or Massachusetts.

### Data collection

All participants completed the study procedures between June 1, 2020 and June 22, 2020. At this point, participants were in a location in which they were entering their second phase of re-opening after initial stay-at-home orders were placed. Participants first completed an online survey, which assessed basic demographics. Following the survey, participants completed semi-structured interviews over the telephone. All interviewers were led by one of two study team members (CP, KD) trained in qualitative methods and each interview followed a structured interview guide. The interview guide (see Supplemental Material) included broad, open-ended questions, which were followed by additional probes, if needed. The current study focused on the dietary behavior questions, which included: (1) Describe your dietary behaviors during the pandemic (Probes were used to explore similarities and differences in eating habits, including alcohol consumption, during the pandemic), (2) What has helped with your eating behaviors?, and (3) What has interfered with your eating behaviors? All interviews were audio-recorded. Interviews were completed until inductive thematic saturation was reached, in which new codes or themes were no longer emerging (Guest et al., 2016; Saunders et al., 2018; Sieidman, 2006). Participants received a $50 gift card for participating in the study.

### Analysis

Descriptive statistics (means and frequencies) were calculated to describe demographic characteristics (e.g. age, race/ethnicity, sex, employment). Audio recordings were transcribed verbatim by a professional transcription company, personal identifiers were removed, and transcripts were imported into Nvivo version 12 for analysis. Following independent reviews of transcripts by three coders and identification of *a priori* and in-vivo themes, initial codes were assigned using content and constant comparative analysis (Glaser, 1965). Coders aimed to build a preliminary taxonomy of codes describing participant responses to our areas of interest: healthy eating barriers and healthy eating facilitators. Additionally, emergent codes and general themes that arose within the data were included. Two coders reviewed each transcript independently, identified codes, and then as a group, each transcript was discussed. A 3rd coder was brought in to resolve any disagreements. The group discussed all identified codes until consensus was met and a mutually agreed upon codebook was finalized.

### Ethics statement

The current qualitative study was reviewed by the University of South Carolina's Institutional Review Board and deemed exempt (Pro00100300); therefore, participants were not required to provide written consent for this secondary study.

## Results

Thirty participants completed the interviews, with durations lasting on average 46.7 ± 10.6 min. Participants were 54.6 ± 10.0 years old, with a body mass index of 31.1 ± 4.5 kg/m^2^ (Table 1). Similar to enrollment in the parent study, the majority of participants were female (83%) and white (87%). Following analysis, several barriers (see section 1 below) and facilitators (section 2) to healthy eating during the early COVID-19 pandemic emerged and are discussed below. Additionally, three general themes (section 3) related to dietary changes emerged including change in eating out frequency or modality, cooking more at home, and changes in alcohol consumption.

**Table 1.** Participant characteristics.

Characteristics (% or mean ± SD)	Participants (*n* = 30)
Age (yrs)	54.6 ± 10.0
Body Mass Index (kg/m^2^)	31.1 ± 4.5
Female	83%
Race Black or African American White Other	3.3% 86.7% 10%
Not Hispanic or Latino	96.7%
Employment Status Full or part time Retired Unemployed	70% 23% 7%
Employment status changed since February 2020	17%
Working from home status Yes No Already working from home before COVID-19 Not working prior to COVID-19	50% 26.7% 3.3% 20%
Children under 18 years in the house	23%

BMI: Body Mass Index.

### Barriers to healthy eating during the initial months of the COVID-19 pandemic

*Snacking/ease of access to food*. The majority of the participants reported an increase in their snacking during the pandemic. Participants stated that because they were not at work or around others, they found themselves snacking and nibbling on food throughout the day. Many individuals also mentioned the availability and temptation of the snacks and how they would work in their kitchen near the refrigerator or cabinets or walk past the cupboards throughout the day. Some participants also mentioned that they found themselves buying more snack food than compared to prior to the pandemic or that because someone else was grocery shopping for them, they noticed an increase in the availability of snack food at home.

In the very beginning, snacking constantly. I sit at my kitchen table to work and it was the refrigerators there, the cabinets are there, there’s no locks on them, no one here to tell me that you shouldn’t be doing that. (ID 706)

Snacking more frequently because I’m here, I’m home (laughs). It’s accessible, where if I was at work eight hours, I only bring what I’m gonna eat, what I have for the day and that’s it. At work, you’re more like, ‘Oh, well I know I have this in the fridge so I can eat, snack on this, snack on that.’ (ID 714)

*Eating as a coping mechanism*. Participants talked a lot about using food as a coping mechanism. Many participants reported an increase in ‘comfort foods’ during the pandemic. Individuals noted they would bake or buy unhealthy foods that they considered to be comfort foods, such as foods high in carbohydrates and sugars, to keep the morale up in their homes and around loved ones or family members. Participants also reported stress or emotional eating and using food as a reward. For example, many participants talked about feeling that they deserved food and would treat themselves because of the bad situation of the pandemic.

I do find that I am more willing to treat myself to something because I feel like, you know, bad situation, treat yourself, if that will make you feel better, then go for it. (ID 704)

I wanna say eat whatever you want to help you through the moment. (ID 728)

Plus, you know what? You kinda feel like you deserve it … Poor us. You know, we’re stuck at home. It’s the poor me mentality. That’s what it is. We deserve to reward ourselves with food. (ID 705)

*Lack of routine/planning*. A common barrier to healthy eating that participants mentioned was the lack of routine and planning in their days. Overall, participants reported a change in the structure of their days (e.g. not attending in person work) and commented that this change influenced their dietary routine as well. Participants reported a lack of keeping track of and writing down what they were eating in addition to no longer being structured with their eating times or portions.

Before I was pretty structured. When I was at work, I would have my breakfast at a certain time. I’d have my lunch at a certain time. I’d go home and have dinner. So, everything was structured … I did my own meals, so I did my own portion sizes and things like that. So, but I’ve gotten away from that now. (ID 713)

I was much more diligent about keeping track of eating. Much more planned eating, writing down, all that stuff. Then holidays and pandemic just that was a slide down. (ID 728)

### Facilitators to healthy eating during the initial months of the COVID-19 pandemic

*Calorie control*. Participants reported that to help stay on track with healthy eating they would control their portion sizes, monitor their calorie intake and try to prepare healthier options when cooking at home. To aid in calorie control, some strategies that participants reported include eating premade meals or meal replacements, weighing and measuring their food to log and count calories, and using the serving size suggested on items or monitoring the size of their portions.

I’ll have to have like Healthy Choice fudgicles or everything has to be portioned in this house, 100 calorie portions. (ID 705)

Pay attention to portion size, and the calorie intake. (ID 720)

*Regular routine/scheduling*. Participants found it to be helpful if they were able to stick to a regular routine or schedule in regard to their eating. For some participants this stayed consistent with what they were doing prior to the pandemic, but for others, they found a new regimen that worked for them. Participants mentioned that sticking to what was a habit for them was helpful and that they tried to keep their schedule as prescribed and regimented as before. Also, many participants commented that meal prepping was helpful for them to remain on a regular eating schedule.

Meal prepping is my main key ‘cause I cook dinner for the family every day, but it’s not necessarily stuff I need to eat (laughs). I meal prep my own then I know I can stay consistent. (ID 714)

Meal planning. When we meal plan, we do it as a family, so my family knows, this is what we’re going to have for dinner and we stick to that plan. That’s a huge thing, because without a plan, I’ll just make a box of pasta, you know. (ID 717)

*Self-monitoring*. Participants found self-monitoring to be helpful when asked about eating healthy and staying on track. Many of the participants reported using phone applications to track their food and a few mentioned keeping food diaries or writing down what they were eating. Although it was unclear whether participants were tracking more or less than before the pandemic, however, of the participants logging food, many commented that the pandemic provided them time to measure and weigh their food to be able to keep accurate logs.

I still keep track of my calories all the time. (ID 708)

I have time to like, I’m actually recording again what I eat. I’m weighing and measuring again. I have time for that … Right now, I have time to do that. I have time to actually journal what I eat. (ID 725)

### Dietary changes during the initial months of the COVID-19 pandemic

*Change in eating out frequency or modality*. Overall, the majority of participants noted a decrease in eating out at restaurants during the pandemic. With restaurants shutting down at the onset of the pandemic, participants mentioned that eating out was simply not an option. However, some participants stated that even as places began to re-open, they were hesitant to eat out or get take-out because they did not want to risk eating food that was prepared and touched by others. At the time of the interviews, many participants reported starting to get take-out food again and talked about the restaurants bringing food out for curbside pickup and/or taking the food to an outdoor area, i.e. a park, to eat. There were also individuals who mentioned a recent experience of dining outdoors at a restaurant. Participants reported wanting to get out of the house, being tired of cooking, and wanting to support restaurants as reasons they started getting take-out or dining outside again. Interestingly, one participant mentioned eating take-out food more during the pandemic as opposed to what their family was doing prior to the pandemic and stated the reason was to support local businesses.

We could order take-out, but we haven’t been out to a restaurant since the pandemic hit. We used to go out on Friday night with friends … we’d meet up and go to a different restaurant in town. And we haven’t been able to do any of that so, the only thing we have been able to order out is typically grinders or pizza or Chinese. (ID 713)

Before, [we’d eat out] sometimes two or three nights a week … But once things started closing down and it was order only, I don’t think we ate out for a month. We did start utilizing take-out again recently, a little bit more so. But again, it’s maybe once a week. (ID 729)

*Cooking more*. Many of the participants reported that they have been cooking more than they were prior to the pandemic. Participants talked about using premade meals and/or getting fast food when they worked or had events for their children in the evenings and how without those obligations, they had time to cook and prepare homemade meals. Participants noted that having more time contributed to cooking more and also cooking new things or ‘experimenting’ with food.

I’m cooking more … I would say I’m home more, I’ve got more time on my hands, so, I cook more. (ID 705)

I cook and a lot of stuff at home … I’m home and I can cook during the day and I can put something in the crock pot or whatever while I’m taking conference calls. (ID 712)

*Changes in alcohol consumption*. A lot of participants talked about changes in the frequency/amount and type of alcohol they consumed, but interestingly, some were increases and some were decreases. There were a few individuals who said the amount of alcohol they consumed did not change, but the types of drinks were different (i.e. drinking less wine and more mixed drink). The reasons participants gave for consuming more alcohol included being stressed as a result of the pandemic and drinking to calm their nerves, drinking while watching tv or the news, and not working or having other obligations. One participant talked about starting to drink earlier in the day, around noon, because they were not at work and how this contributed to an increase in alcohol consumption. There were also participants who reported consuming less alcohol than prior to the pandemic. The most common reason for less alcohol consumption was that participants considered themselves ‘social drinkers’ and said the lack of social activities and engagements led to them not drinking during the pandemic.

I think I definitely have been drinking a little more than usual, or more frequently is probably a better word. I don’t know that it’s more, but it’s more regularly. I think, you know, that’s just all escapism. (ID 710)

I quit drinking. I don’t, I only drink once a week. ‘Cause I only drink during social situations and I haven’t socialized that much. (ID 725)

## Discussion

This study explored both the challenges and facilitators of healthy eating during the early months (March-June) of the COVID-19 pandemic among a group of individuals enrolled in an internet-based weight loss program. Barriers that emerged included snacking/ease of access to food, eating as a coping mechanism, and lack of routine. Facilitators included controlling caloric intake, having a regular routine and planning, and self-monitoring. Participants also spoke about a variety of changes in their eating behaviors, some of which were problematic, and others that were quite positive.

One of the most common barriers mentioned by participants was snacking and ease of access to food. Due to lockdowns and other factors, people spent more time at home (50% of the sample were working from home), which provided many more opportunities for snacking. For example, participants described working in their kitchen and having more snack food around and in closer proximity than usual either due to their purchases or someone else shopping on their behalf. This result is consistent with other research suggesting people increased their snacking during the pandemic (Ammar et al., 2020). Snacking in between meals on healthy foods could be beneficial to health and weight, (Almoraie et al., 2021) however, many snack choices are high in calories (Barnes et al., 2015). As a result, constant availability of unhealthy foods paired with frequent eating episodes could lead to increased caloric consumption, (Kant, 2014) which would be problematic for individuals attempting to lose weight, such as those in the current study. Although stay-at-home orders have been lifted, many employers are still allowing employees to work from home, which means snacking and ease of access to food may continue to be a significant challenge for many adults. Future weight loss programs and public health campaigns may consider providing additional tips to overcome this barrier.

The importance of having a regular routine and the ability to meal plan was evident as it emerged both as a barrier and facilitator to healthy eating. Participants described a major change in their daily schedule as they were no longer working away from home, which led to less structure regarding their eating routine and negatively influenced when and how much they ate. On the other hand, some participants commented on how sticking to a schedule or regular routine with their eating despite experiencing changes to their day and employment, was helpful. Previous research focusing on barriers and facilitators to physical activity found similar results in that changes in daily routines either can be helpful or interfere with participating in regular physical activity (Petersen et al., 2021; Roche et al., 2022)., Planning meals or times to engage in physical activity could be an important strategy to emphasize within lifestyle interventions, as it would likely remain beneficial even beyond the COVID-19 pandemic, as unexpected circumstances or major life events can frequently disrupt daily routines. Interventions could focus on teaching strategies to create new routines immediately after major life disruptions, methods for staying consistent with routines despite busy or stressful weeks, or strategies for how to make meal planning a priority during all seasons of life.

Participants also discussed how calorie control and dietary self-monitoring were facilitators of healthy eating during the pandemic. Both of these techniques are common behavioral strategies encouraged during weight loss programs, (Butryn et al., 2011) including the current internet-based program that participants were enrolled in (Unick et al., 2020). Despite facing challenges from changes in their daily routine, many participants indicated they had more time during the pandemic to weigh or measure their food, as well as to track their diet. Dietary self-monitoring is highly burdensome, however, it is well established that frequent and consistent self-monitoring is associated with weight loss (Burke et al., 2011). The use of technology can help to reduce some of the burden of self-monitoring, but at the same time, if it is too easy, it may be hindering habit formation and engagement (Turner-McGrievy et al., 2021). Future research is needed to identify strategies and self-monitoring techniques that are less burdensome, yet can still keep users engaged and adherent to daily dietary tracking, even when faced with stressful situations or changes in their routine.

Unfortunately, many participants spoke about using food and alcohol as ways to cope with the stress of the pandemic. An increase in the consumption of ‘comfort foods,’ which are high in carbohydrates and sugar, were specifically mentioned by many participants. These ‘comfort foods’ were used as a reward, to improve mood, and to lower stress. This finding is not surprising given reports of lower physical activity (Stockwell et al., 2021) and increased incidence of stress, depression, and anxiety during the pandemic (Salari et al., 2020). Although some participants described consuming less alcohol than before the pandemic because they were ‘social drinkers,’ others reported an increase in alcohol consumption due to feeling stressed or having less obligations. The individual variations in consumption during the pandemic are similar to previous research on alcohol consumption, (Rossow et al., 2021) as well as other addictive behaviors (Hodgins & Stevens, 2021; Roberts et al., 2021). The increase in alcohol not only may hinder weight management, (Kase et al., 2016) but could also further exacerbate levels of anxiety and depression (Boden & Fergusson, 2011). Future weight management programs may want to place increased emphasis on healthy ways to cope with stress, boredom, and other emotional triggers, in order to reduce consumption of comfort foods and alcohol during high-risk periods.

This study had both strengths and limitations. A major strength of the current study is that it provides an in-depth look at the factors helping and hindering healthy eating during the pandemic among individuals actively seeking to lose weight. The results can help to inform modifications to current behavioral weight loss programs or public health recommendations to help overcome barriers and promote facilitators of healthy eating during the ongoing pandemic. They can also inform future interventions so that weight loss seeking individuals are better equipped to overcome some of these identified barriers when other life disruptions occur. Limitations of the study include having a predominately White female sample from the same geographical location and weight loss program. There is evidence to suggest that food preferences and eating habits differ based on sex (Modlinska et al., 2020) and race; (Thompson et al., 2020) thus, it is likely that barriers and facilitators of healthy eating differed in other locations and populations. Further, participants were enrolled in the weight loss program for 4–11 months and responses may differ based on duration in program. Finally, participants were only interviewed in the early months of the pandemic which limits any understanding about changes in barriers and facilitators over time.

In closing, this study suggests that spending more time at home was linked to both positive (e.g. eating schedule, cooking at home, eating healthy food) and negative (e.g. more snacking, lack of an eating schedule, eating more food than usual) eating behaviors. More importantly, even in the midst of a life-altering pandemic, individuals were able to continue to use what they were learning/had learned (e.g. calorie control techniques, self-monitoring, scheduling of eating) during an internet-based weight loss program. As the pandemic is still ongoing, future studies should examine whether eating behaviors continue to change. Additionally, as there was variability in barriers and facilitators experienced, more research is needed on how best to tailor behavioral weight loss programs and public health recommendations based on individual differences and preferences.

## Supplementary Material

Supplemental Material
