# Supplementary material for: Bad situation, treat yourself: a qualitative exploration of the factors influencing healthy eating habits during the COVID-19 pandemic
Source: Health Psychol Behav Med. 2023 Mar 2;11(1):2182307. doi: 10.1080/21642850.2023.2182307 (PMC9987739; doi:10.1080/21642850.2023.2182307)
Supplement: Supplemental Material [file RHPB_A_2182307_SM5116.docx]

**Influence of the COVID-19 Pandemic on Stress and Dietary and Physical Activity Behaviors:**

**A Qualitative Investigation**

Interview Guide

Principal Investigator:

Christine A. Pellegrini, PhD

Department of Exercise Science

803-777-0911

cpellegrini@sc.edu

Funded by:

UofSC COVID-19 Research Initiative

Version Number:

V1

Version Date:

May 14, 2020

Contents

[Welcome & introductions (<5 minutes) 3](#_Toc40281855)

[Purpose (5 minutes) 3](#_Toc40281856)

[Conclusion of the Interview (<5 minutes) 3](#_Toc40281857)

[**Interview Protocol:** 4](#_Toc40281858)

[General Experiences (15 minutes) 4](#_Toc40281859)

[Dietary Behaviors (10 minutes) 5](#_Toc40281860)

[Physical Activity and Sedentary Behavior (20 minutes) 6](#_Toc40281861)

[Weight Loss and Motivation (5 minutes) 8](#_Toc40281862)

## Welcome & introductions (<5 minutes)

*Thanks for agreeing to be part of our study today. We appreciate your willingness to share your thoughts with us about stress, diet, physical activity, and weight loss during the COVID19 pandemic.*

[Interviewer introduction]

## Purpose (<5 minutes)

*We are conducting interviews to understand how the COVID-19 pandemic has influenced your everyday life and behaviors. Specifically, we want to understand how societal and policy changes have influenced you and how those changes may have influenced with your eating and physical activity behaviors. We want to understand how different individuals react during this stressful situation and discuss coping strategies that you may have used that have helped with weight management. We hope that by learning how people have been affected and how they have dealt with the challenges will help us to better inform and tailor future weight management programs and public health recommendations not only in the event of a future pandemic, but also for everyday stressful life events.*

*Before we get started, I just want to remind you that there are no right or wrong answers. Every person’s experiences and opinions are important and we hope to hear a wide range of viewpoints. This interview will be audio recorded, however, your answers will be kept confidential. You are free to skip any questions that make you feel uncomfortable. Do you have any questions?*

[Conduct Interview Using Interview Guide Below]

## Conclusion of the Interview (<5 minutes)

*I will now end the recording. Thank you for completing the interview and the surveys. We will start processing your $50 and you should receive it soon. Please let me know if you have any questions.*

# **Interview Protocol:**

## General Experiences (15 minutes)

*First I’m going to discuss general experiences with the COVID-19 pandemic*

1. Please tell me how the COVID-19 pandemic has been for you?

►PROBES:

1. What specifically has changed for you?

- Employment, work obligations, and conditions (e.g., working from home)?
- Financial changes?
- Changes in childcare?
- Typical routine?

1. How have things changed since when it started (March 2020) until now?
2. How have you handled any changes?
3. If at all, how has the pandemic affected your mental well-being you?

►PROBES:

1. What specifically has changed for you?

- Stress?
- Anxiety?
- Depression?
- Sleep disturbance?

1. How have things changed since when it started (March 2020) until now?
2. How have you handled any changes?
3. If at all, how has the pandemic physically affected you?

►PROBES:

1. What specifically has changed for you?

- Headaches?
- Gastrointestinal changes?
- Weight gain?

1. How have things changed since when it started (March 2020) until now?
2. How have you handled any changes?
3. To what extent do you think positively/negatively about the present?
4. To what extent do you think positively/negatively about the future?

## Dietary Behaviors (10 minutes)

*Next, I want to shift gears a bit and ask you some questions about your dietary behaviors during the pandemic.*

1. Describe your dietary behaviors during the pandemic?

►PROBES:

1. What has been DIFFERENT with your dietary behaviors since the pandemic?
   - Snacking?
   - Ability to eat out?
   - Ability to access food?
   - Alcohol consumption?
2. What has been the SAME with your dietary behaviors since the pandemic?
3. How have things changed since when it started (March 2020) until now?
4. What has helped you with your eating behaviors?

►PROBES:

1. (intrapersonal) – How have personal factors (e.g., your motivation, knowledge, age, self-monitoring) helped?
2. (interpersonal) - How have others (e.g., family members, coworkers, neighbors) helped, or not being around others helped?
3. (organizational/institutional) – How have institutional/organizational (e.g., worksites, schools, childcare) rules and policies helped?
4. (community) – How has your community or environment helped?
5. (policy) – How have local, state, and national policies (e.g., closing of indoor dining) helped?
6. What has interfered with your eating behaviors?

►PROBES:

1. (intrapersonal) – How have personal factors (e.g., your motivation, knowledge, age) interfered?
2. (interpersonal) - How have others (e.g., family members, coworkers, neighbors) helped, or not being around others interfered?
3. (organizational/institutional) – How have institutional/organizational (e.g., worksites, schools, childcare) rules and policies interfered?
4. (community/environment) – How has your community or environment interfered?
5. (policy) – How have local, state, and national policies (e.g., closing of indoor dining) interfered?
6. What do you think your eating behaviors will look like in the future, after the pandemic?

## Physical Activity and Sedentary Behavior (20 minutes)

*Next, I want to shift gears a bit and ask you some questions about your physical activity behaviors during the pandemic.*

1. Describe your physical activity and exercise during the pandemic

►PROBES:

1. What has been DIFFERENT with your physical activity and exercise since the pandemic?
   - Belong to a gym that closed?
   - Unable to go outside?
2. What has been the SAME with your physical activity and exercise since the pandemic?
3. How have things changed since when it started (March 2020) until now?
4. What has helped you with your physical activity and exercise?

►PROBES:

1. (intrapersonal) – How have personal factors (e.g., your motivation, knowledge, age, use of a physical activity monitor/self-monitoring) helped?
2. (interpersonal) - How have others (e.g., family members, coworkers, neighbors) helped, or not being around others helped?
3. (organizational/institutional) – How have institutional/organizational (e.g., worksites, schools, childcare) rules and policies helped?
4. (community) – How has your community or environment helped?
5. (policy) – How have local, state, and national policies helped?
6. What has interfered with your physical activity and exercise?

►PROBES:

1. (intrapersonal) – How have personal factors (e.g., your motivation, knowledge, age) interfered?
2. (interpersonal) - How have others (e.g., family members, coworkers, neighbors) helped, or not being around others interfered?
3. (organizational/institutional) – How have institutional/organizational (e.g., worksites, schools, childcare) rules and policies interfered?
4. (community/environment) – How has your community or environment interfered?
5. (policy) – How have local, state, and national policies (e.g., closing of gyms & parks, stay at home orders) interfered?
6. What do you think your physical activity will look like in the future, after the pandemic?

*Next, I want to shift gears again a little bit and ask you some questions about the time you spend sitting.*

1. Describe your sedentary behavior or time you spent sitting during the pandemic

►PROBES:

1. What has been DIFFERENT with your sitting time since the pandemic?
   - More screen/leisure time?
   - More computer/work time?
2. What has been the SAME with your sitting time since the pandemic?
3. How have things changed since when it started (March 2020) until now?
4. What do you think your sitting time will look like in the future, after the pandemic?
5. What has helped you sit less?

►PROBES:

1. (intrapersonal) – How have personal factors (e.g., your motivation, knowledge, age, own a stand up desk) helped?
2. (interpersonal) - How have others (e.g., family members, coworkers, neighbors) helped, or not being around others helped?
3. (organizational/institutional) – How have institutional/organizational (e.g., worksites, schools, childcare) rules and policies helped?
4. (community) – How has your community or environment helped?
5. (policy) – How have local, state, and national policies helped?
6. What has made you sit more?

►PROBES:

1. (intrapersonal) – How have personal factors (e.g., your motivation, knowledge, age) interfered?
2. (interpersonal) - How have others (e.g., family members, coworkers, neighbors) helped, or not being around others interfered?
3. (organizational/institutional) – How have institutional/organizational (e.g., worksites, schools, childcare) rules and policies interfered?
4. (community/environment) – How has your community or environment interfered?
5. (policy) – How have local, state, and national policies interfered?

## Weight Loss and Motivation (5 minutes)

*Finally, I want to discuss your weight and changes in motivation since starting the XXX program/study.*

1. Please tell me about your initial motivation to lose weight and join the XXX program/study?
2. BEFORE the pandemic, on a scale of 0 (not at all important) to 10 (very important), how important was weight loss to you?
3. DURING the start of the pandemic, on a scale of 0 (not at all important) to 10 (very important), how important was weight loss to you?
4. NOW, on a scale of 0 (not at all important) to 10 (very important), how important is weight loss to you?
5. Why has the importance of weight loss changed/stayed the same?

Any other comments?
